# Transethnic meta-analysis of rare coding variants in *PLCG2*, *ABI3*, and *TREM2* supports their general contribution to Alzheimer’s disease

**DOI:** 10.1038/s41398-019-0394-9

**Published:** 2019-01-31

**Authors:** Maria Carolina Dalmasso, Luis Ignacio Brusco, Natividad Olivar, Carolina Muchnik, Claudia Hanses, Esther Milz, Julian Becker, Stefanie Heilmann-Heimbach, Per Hoffmann, Federico A. Prestia, Pablo Galeano, Mariana Soledad Sanchez Avalos, Luis Eduardo Martinez, Mariana Estela Carulla, Pablo Javier Azurmendi, Cynthia Liberczuk, Cristina Fezza, Marcelo Sampaño, Maria Fierens, Guillermo Jemar, Patricia Solis, Nancy Medel, Julieta Lisso, Zulma Sevillano, Paolo Bosco, Paola Bossù, Gianfranco Spalletta, Daniela Galimberti, Michelangelo Mancuso, Benedetta Nacmias, Sandro Sorbi, Patrizia Mecocci, Alberto Pilotto, Paolo Caffarra, Francesco Panza, Maria Bullido, Jordi Clarimon, Pascual Sánchez-Juan, Eliecer Coto, Florentino Sanchez-Garcia, Caroline Graff, Martin Ingelsson, Céline Bellenguez, Eduardo Miguel Castaño, Claudia Kairiyama, Daniel Gustavo Politis, Silvia Kochen, Horacio Scaro, Wolfgang Maier, Frank Jessen, Carlos Alberto Mangone, Jean-Charles Lambert, Laura Morelli, Alfredo Ramirez

**Affiliations:** 1 Laboratory of Amyloidosis and Neurodegeneration, Fundación Instituto Leloir-IIBBA-CONICET, Ciudad Autónoma de Buenos Aires (C.A.B.A.), Buenos Aires, Argentina; 2 Centro de Neuropsiquiatría y Neurología de la Conducta (CENECON), Facultad de Medicina, Universidad de Buenos Aires (UBA), C.A.B.A, Buenos Aires, Argentina; 3 Departamento Ciencias Fisiológicas UAII, Facultad de Medicina, UBA, C.A.B.A, Buenos Aires, Argentina; 4 Hospital Interzonal General de Agudos Eva Perón, San Martín, Buenos Aires, Argentina; 5 Laboratorio de Bioquímica Molecular, Facultad de Medicina, Instituto de Investigaciones Médicas A. Lanari, UBA, C.A.B.A, Buenos Aires, Argentina; 6 Department of Psychiatry and Psychotherapy, University of Bonn, 53127 Bonn, Germany; 7 Division of Neurogenetics and Molecular Psychiatry, Department of Psychiatry and Psychotherapy, University of Cologne, 50937 Cologne, Germany; 8 Institute of Human Genetics, University of Bonn, School of Medicine & University Hospital, 53127 Bonn, Germany; 9 Department of Genomics, Life & Brain Center, University of Bonn, 53127 Bonn, Germany; 10 Division of Medical Genetics, University Hospital and Department of Biomedicine, University of Basel, CH-4058 Basel, Switzerland; 11 Ministerio de Salud de la Provincia de Jujuy, Programa del Adulto Mayor, San Salvador de Jujuy, Jujuy, Argentina; 12 Neurosciences and Complex Systems Unit (EnyS), CONICET, Hospital El Cruce “Dr. Néstor Kirchner”, Univ Arturo Jauretche, F. Varela, Buenos Aires, Argentina; 13 Instituto di Ricovero e Cura a Carattere Scientifico (IRCCS) Associazione Oasi Maria Santissima Srl, Troina, Italy; 14 Department of Clinical and Behavioural Neurology, Experimental Neuropsychobiology Laboratory, Rome, Italy; 15 Department of Clinical and Behavioural Neurology, Neuropsychiatry Laboratory, IRCCS Santa Lucia Foundation, Rome, Italy; 16 Neurodegenerative Diseases Center, University of Milan, Centro Dino Ferrari, Fondazione Ca′ Granda, IRCCS Ospedale Maggiore Policlinico, Milan, Italy; 17 Department of Experimental and Clinical Medicine, Neurological Institute, University of Pisa, Pisa, Italy; 18 NEUROFARBA (Department of Neuroscience, Psychology, Drug Research and Child Health), University of Florence, Florence, Italy; 19 IRCCS ′Don Carlo Gnocchi′, Florence, Italy; 20 Section of Gerontology and Geriatrics, Department of Medicine, University of Perugia, Perugia, Italy; 21 Geriatric Unit and Gerontology-Geriatrics Research Laboratory, Department of Medical Sciences, IRCCS Casa Sollievo della Sofferenza, San Giovanni Rotondo, Italy; 22 Section of Neuroscience, DIMEC, University of Parma, Parma, Italy; 23 Alzheimer Center, FERB, Gazzaniga, Bergamo, Italy; 24 Neurodegenerative Disease Unit, Department of Basic Medicine, Neuroscience, and Sense Organs, University of Bari Aldo Moro, Bari, Italy; 25 Instituto de Investigación Sanitaria Hospital la Paz (IdiPAZ), Madrid, Spain; 26 Centro de Investigación Biomédica en Red de Enfermedades Neurodegenerativas (CIBERNED), Instituto de Salud Carlos III, Madrid, Spain; 27 Centro de Biología Molecular Severo Ochoa (CSIC-UAM), Madrid, Spain; 28 Memory Unit, Neurology Department and Sant Pau Biomedical Research Institute, Hospital de la Santa Creu i Sant Pau, Autonomous University of Barcelona, Barcelona, Spain; 29 Neurology Service and CIBERNED, ′Marqués de Valdecilla′ University Hospital (University of Cantabria and IDIVAL), Santander, Spain; 30 Molecular Genetics Laboratory-Hospital, University of Central Asturias, Oviedo, Spain; 31 Immunology Service, Hospital Universitario de Gran Canaria Doctor Negrín, Las Palmas de Gran Canaria, Spain; 32 Genetics Unit, Theme Aging, Karolinska University Hospital, Solna, Sweden; 33 Division of Neurogeriatrics, Department NVS, Karolinska Institutet, Bioclincum J10:20, Solna, Sweden; 34 Department of Public Health/Geriatrics, Uppsala University, Uppsala, Sweden; 35 INSERM, U1167, RID-AGE-Risk Factors and Molecular Determinants of Aging-Related Diseases, F-59000 Lille, France; 36 Institut Pasteur de Lille, F-59000 Lille, France; 37 University Lille, U1167-Excellence Laboratory LabEx DISTALZ, F-59000 Lille, France; 38 German Center for Neurodegenerative Diseases (DZNE), 53127 Bonn, Germany; 39 Department of Neurodegenerative Diseases and Geriatric Psychiatry, University of Bonn, 53127 Bonn, Germany; 40 Department of Psychiatry and Psychotherapy, University of Cologne, 50937 Cologne, Germany

## Abstract

Rare coding variants in *TREM2*, *PLCG2*, and *ABI3* were recently associated with the susceptibility to Alzheimer’s disease (AD) in Caucasians. Frequencies and AD-associated effects of variants differ across ethnicities. To start filling the gap on AD genetics in South America and assess the impact of these variants across ethnicity, we studied these variants in Argentinian population in association with ancestry. *TREM2* (rs143332484 and rs75932628), *PLCG2* (rs72824905), and *ABI3* (rs616338) were genotyped in 419 AD cases and 486 controls. Meta-analysis with European population was performed. Ancestry was estimated from genome-wide genotyping results. All variants show similar frequencies and odds ratios to those previously reported. Their association with AD reach statistical significance by meta-analysis. Although the Argentinian population is an admixture, variant carriers presented mainly Caucasian ancestry. Rare coding variants in *TREM2*, *PLCG2*, and *ABI3* also modulate susceptibility to AD in populations from Argentina, and they may have a European heritage.

## Introduction

Alzheimer’s disease (AD) is the most common form of dementia, and has an estimated genetic component of 60–80%^1^. Over the last decade, more than 20 loci containing common genetic variants (minor allele frequency (MAF) >5%) have been associated with AD^2^. The advent of new genetic sequencing technologies has enabled the identification of several rare variants (MAF <1%) with moderate effects on AD susceptibility^3^. In 2017, the International Genomics of Alzheimer’s Project (IGAP) reported four rare coding variants significantly associated with AD^4^, all of them being involved in microglial-mediated innate immunity. Two of them are novel nonsynonymous variants: a protective one in *PLCG2* (rs72824905) and a risk one in *ABI3* (rs616338). The other two were previously reported in the susceptibility gene *TREM2* (rs143332484 and rs75932628), and are responsible for p.R62H and p.R47H substitutions, respectively. The findings in *TREM2* have been consistently replicated in Caucasian^4–6^ and African-American populations^7^. However, the association of *TREM2* p.R47H with AD could not be found in East Asian population, because its frequency is extremely low^8,9^. This latter observation suggests ethnic variability fostering investigation of these variants in different ethnic groups. Given the increasing population diversity observed in countries all over the world, understanding population-shared and -specific risk factors of AD will translate into improved and specific prevention and/or treatment for people.

Latin America is a vast territory, with a wide diverse admixture of European, Native American, and African ancestral populations. The genetic architecture of sporadic AD has not been studied in this population beyond *APOE-ε4*. In this report, we provide the first evidence for an association between *TREM2*, *PLCG2*, and *ABI3* rare variants and AD in the Argentinian population.

## Methods

### Subjects

Individuals with AD and without cognitive impairment older than 60 years were recruited from outpatient Neurology Departments of the following hospitals: Instituto de Investigaciones Médicas “Alfredo Lanari” and Hospital de Clínicas (Buenos Aires City), Hospital Interzonal General de Agudos “Eva Perón” (General San Martín county), Hospital El Cruce “Dr. Néstor Kirchner” (Florencio Varela county), and several assistance centers located across Jujuy province. Only samples from people born in Argentina or in South America were included in the analysis. This study was approved by the ethical committee “Comité de Bioética Fundación Insituto Leloir (HHS IRB #00007572, IORG # 006295, FWA00020769)” by the approval of protocol CBFIL #22. All participants and/or family members gave their informed consent.

Diagnosis of AD followed diagnostic criteria from the National Institute of Neurological and Communicative Disorders and Stroke and the Alzheimer’s disease and Related Disorders Association (NINCDS-ADRDA)^10^. The diagnosis of AD includes a clinical examination to evaluate functionality and activities of daily living that should be compromised; a complete panel of neurocognitive tests to evaluate memory, attention, language, and executive function, of which one or more should be altered; a computerized tomography and/or magnetic resonance imaging to assess cortical–hippocampal atrophy and vascular events, and a blood test analysis to exclude metabolic or infectious causes of dementia. Individuals were included as controls if neurocognitive and clinical assessments were normal.

### Genotyping and statistics

Genomic DNA was isolated using standard procedures from whole blood or saliva samples. *TREM2* (rs143332484 and rs75932628), *PLCG2* (rs72824905), and *ABI3* (rs616338) variants were genotyped using custom-designed TaqMan assays (Thermo Fisher). Assay accuracy was checked by including positive and negative controls in each experiment. *APOE* alleles were determined by genotyping rs429358 and rs7412. Association with AD was calculated using Fisher’s exact test with statistical significance of *p* < 0.05. All variants were in Hardy–Weinberg equilibrium (HWE, *p* > 0.05). Power calculations were performed using Genetic Power Calculator for discrete traits (http://zzz.bwh.harvard.edu/gpc/cc2.html). Meta-analysis for the effect of rare variants in association with AD was conducted using beta and standard error with Metafor R-package^11^. Populations from France (*n* = 8514), Italy (*n* = 2306), Spain (*n* = 3966), and Sweden (*n* = 2286) from the European Alzheimer's Disease Initiative (EADI)^4^ were included in the meta-analysis.

### Ancestry of the population

European (CEU, *n* = 85), Yorubas African (AFR, *n* = 88), and Native American (NAM, *n* = 46) ancestral populations were obtained from 1000 Genomes (http://www.internationalgenome.org/). Argentinian samples (ARG) were subjected to genome-wide genotyping using the Infinium Global Screening Array (GSA) v.1.0+GSA shared custom content (Illumina). Quality controls (QC) were performed as described before^12^, using PLINK v1.9^13^ and R v3.4.4^14^. After QC, remaining samples (*n* = 834) have <5% of missing genotypes and passed sex-check and identity-by-state filters. Remaining single nucleotide polymorphisms (SNPs) have >95% call rate, MAF >1%, are in HWE (*p* > 10^−6^), and without differences in call rate between cases and controls (*p* < 1 × 10^−5^).

For the ancestry analyses, overall population ancestry was first evaluated for ARG by extracting 446 ancestry informative markers (AIMs), which were specifically selected to estimate ancestry in Latin America^15^, from the genotyped data. Second, ancestry of chromosomes containing rare variants was evaluated by extracting from the 446 AIMS, the AIMs in chromosome 6 from people carrying *TREM2* p.R47H and *TREM2* p.R62H, those AIMs in chromosome 16 from people carrying *PLCG2* p.P522R, and finally AIMs in chromosome 17 from people carrying *ABI3* p.S209F. For each ancestry estimation, the same AIMs were extracted from CEU, AFR, and NAM. Ancestry was predicted using ADMIXTURE v1.3.0^16^.

## Results

To evaluate the association of the four rare variants recently reported by IGAP^4^ with AD in an Argentinian population sample (ARG), 905 participants were recruited from different regions of the country. Demographic and clinical information of the 419 AD cases and 486 controls is summarized in Table 1. We first explored the risk effect of *APOE-ε4* allele on AD susceptibility confirming thereby previous reports (odds ratio (OR) = 3.14, *p* < 0.0001)^17^. For *APOE-ε2*, we observed the expected protective effect, although it did not reach statistical significance (OR = 0.77, *p* < 0.33). Next, we genotyped the recently described rare variants^4^, i.e., *TREM2* p.R47H (rs75932628) and p.R62H (rs143332484), *PLCG2* p.P522R (rs72824905) and *ABI3* p.S209F (rs616338). All of them were detected in ARG with MAFs similar to those reported by IGAP (Table 2)^4^. They also showed similar magnitude of association, even though neither of these variants reached statistical significance (Table 2)^4^. This observation was expected, since our sample had a power of 60% to detect OR = 3 of a variant with MAF = 0.01. Notwithstanding, the fact that all variants showed similar effect sizes and effect directions as in the report of IGAP prompted us to perform a meta-analysis using samples from France, Italy, Spain, and Sweden (EADI)^4^. Despite larger sample size, statistical power associated with EADI does not allow to reach nominal significant association with AD risk. However, when meta-analyzing both EADI and ARG samples, this gain in power is enough to help in reaching statistical significance for the variants analyzed, in particular the *TREM2* p.R47H variant (Table 3, Figure S1). Unfortunately, information for *TREM2* p.R62H was not available. This can be explained by the observation that the variant effects in ARG are similar to those detected in the other European populations analyzed (as indicated by heterogeneity statistic (*I*^*2*^), see Table 3). All these results together support the hypothesis that these rare variants are also associated to AD in ARG at a similar level than the one observed in Europe.

**Table 1:** Argentinian sample demographics

	No. of subjects (female %)	Age (years)		AAO mean (SD)	MMSE mean (SD)	CDR mean (SD)	*APOE* freq (%)			*APOE-*ε4 carriers (%)
		Mean (SD)	Range				ε2	ε3	ε4	
Cases	419 (64.4)	77.2 (6.3)	62–96	72.5 (6.5)	18.3 (5.8)	1.4 (0.75)	3.5	69.5	27.0	45.6
Controls	486 (65.6)	74.6 (7.5)	59–105		28.5 (1.2)	0.3 (0.3)	5.5	84.2	10.4	19.6

*SD* standard deviation, *AAO* age at onset, *MMSE* Mini-Mental State Examination, *CDR* Clinical Dementia Rating scale, *APOE freq* apolipoprotein E allele frequency

**Table 2:** Genotyping results for *TREM2*, *PLCG2*, and *ABI3*

Gene	Protein variation	MAF cases	MAF controls	Allele cases	Alleles controls	OR	95% CI	*P* value	OR_IGAP_
*TREM2*	p.R47H	0.005	0.001	4|816	1|948	4.68	0.46–230.84	0.19	2.46
*TREM2*	p.R62H	0.012	0.009	10|816	9|956	1.31	0.47–3.68	0.64	1.67
*PLCG2*	p.P522R	0.004	0.006	3|810	6|944	0.58	0.09–2.73	0.52	0.68
*ABI3*	p.S209F	0.012	0.004	9|772	4|912	2.70	0.75–12.07	0.10	1.43

*MAF* minor allele frequency, *OR* odds ratio, *CI* confidence interval, *IGAP* International Genomics of Alzheimer’s Project

**Table 3:** Contribution of Argentinian samples to meta-analysis

Gene	Protein variation	Populations	OR	95% CI	*P* value	*I* ^2^
*TREM2*	p.R47H	EADI	2.10	1.04–4.27	0.04	0.00
		EADI+ARG	2.29	1.17–4.47	0.02	0.00
*PLCG2*	p.P522R	EADI	0.60	0.35–1.03	0.06	4.17
		EADI+ARG	0.60	0.36–0.99	0.05	0.00
*ABI3*	p.S209F	EADI	1.49	0.90–2.48	0.12	0.00
		EADI+ARG	1.58	0.98–2.57	0.06	0.00

*OR* odds ratio, *CI* confidence interval, *I*^*2*^ heterogeneity statistic

**Fig. 1:**
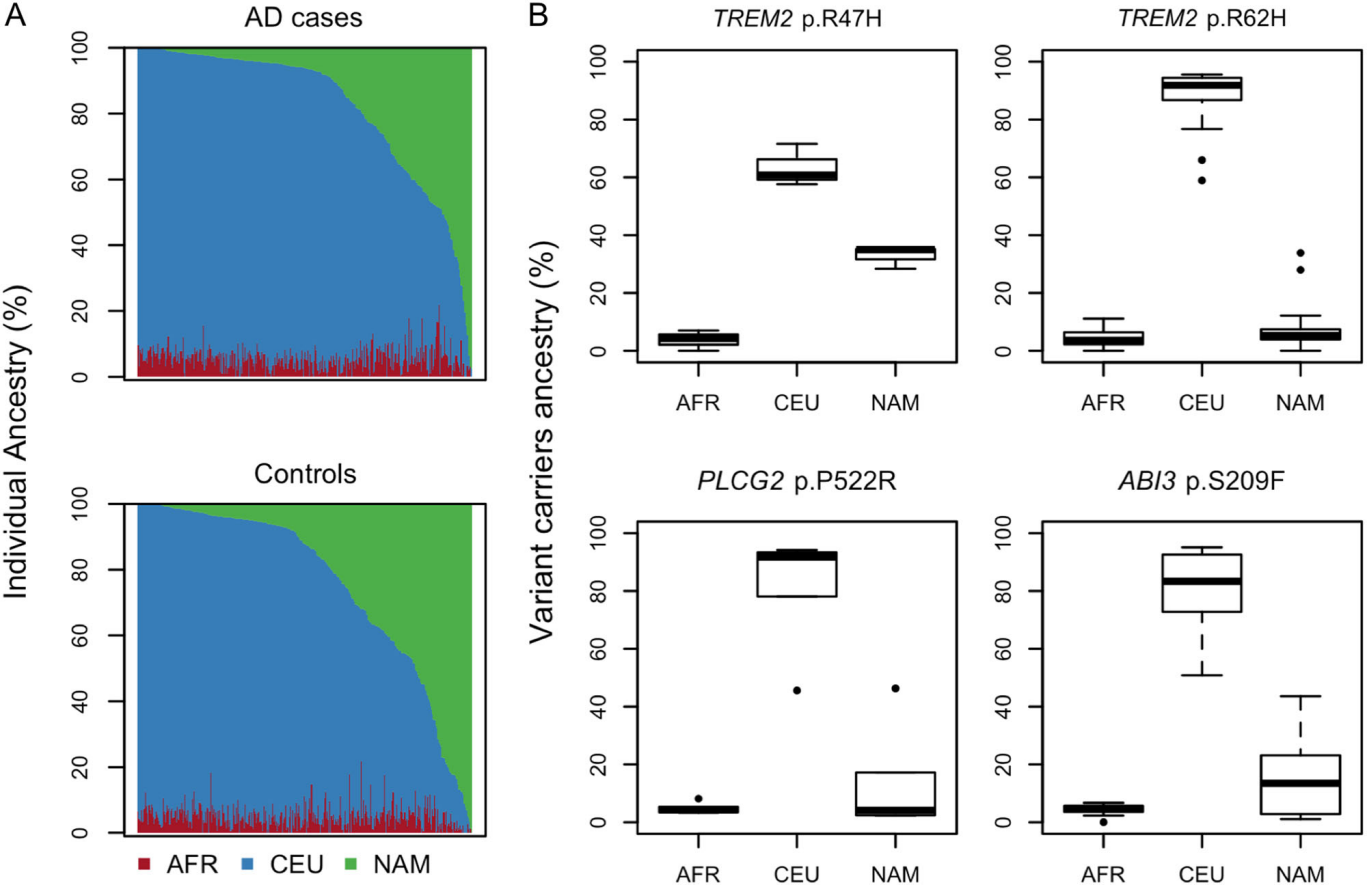
Ancestry analysis of DNA samples that passed quality controls. **a** Distribution of genetic ancestry in Alzheimer’s disease (AD) cases and controls. Bar-plots represent each participant on the *x*-axis, and his percent of European (CEU), African (AFR), and Native American (NAM) ancestry on the *y*-axis. **b** Ancestry of rare variant carriers. Box-plots show ancestry composition in percent of people carrying *TREM2* p.R47H (*n* = 3), *TREM2* p.R62H (*n* = 16), *PLCG2* p.P522R (*n* = 6), and ABI3 p.S209F (*n* = 11) mutations

**Fig. 2:**
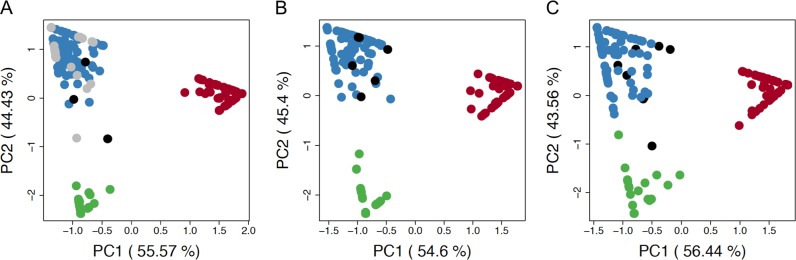
Ancestry of chromosomes containing the rare variants. Principal component analysis (PCA) of ancestry results for **a** chromosome 6, containing *TREM2* p.R47H (black) and *TREM2* p.R62H (gray); **b** chromosome 16, containing *PLCG2* p.P522R (black); and **c** chromosome 17, containing *ABI3* p.S209F. Ancestral populations are European (blue), African (red), and Native American (green). Percent of distribution explained by each principal component (PC) it is shown in parenthesis

Although it is generally accepted that the population from Argentina is mostly originated from Europe, several studies have shown that this population is an admixture of predominantly European and Native American ancestry^18^. To estimate the ancestry of ARG, we used a panel of 446 SNPs, reported to be precisely balanced to study Latin American populations^15^. ARG showed to be an admixture of mainly CEU and NAM, and to a lesser extent of AFR (Fig. S2). Proportions of ancestries are equally distributed among cases and controls (Fig. 1a), indicating that association analysis may not be biased by population stratification. Furthermore, we looked at the ancestry of people carrying the rare variant mutations in ARG (Fig. S3) and detected that CEU component was predominant in all the carriers (Fig. 1b). Although ancestry estimation at the specific locus was not possible due to the lack of AIMs in close proximity to the rare variants, the ancestry of the chromosomes containing the rare variants showed to be CEU (Fig. 2), suggesting a European heritage for the studied variants.

## Discussion

Here we show information from a case–control study performed in Argentina, being the first study on sporadic AD genetics in South America, beyond *APOE*. Our results strongly suggest that rare coding variants described by IGAP in *TREM2*, *PLCG2*, and *ABI3* might also modulate the susceptibility to AD in this population. In addition, we confirmed, as previously reported^18^, that ARG is an admixture of mainly NAM and CEU. NAM ancestry stemmed from the first settlers of the Americas, who originated from an East Asian population that migrated from Siberia^19^. On the other hand, CEU ancestry is a consequence of the pro-immigration legislation to populate Argentina during nineteenth–twentieth century^20^. In this context, we observed that while *APOE-ε4* OR was similar to that reported for Caucasians (OR_ARG_ = 3.14 vs OR_CEU_ = 3.6, http://www.alzgene.org/), its frequency in AD cases was lower (26.9% in ARG vs. 38% in CEU, http://www.alzgene.org/). This lower frequency is in agreement with data previously reported in other Latin American countries^21^. Interestingly, it is among the lowest worldwide together with that of Mediterranean basin and Native Americans^22,23^, which are the main contributors to Argentinian admixture.

It is of note that the rare variants studied here showed MAFs similar to those reported by IGAP in Caucasians^4^. Unfortunately, since there are no reports in Amerindians, we could not compare their MAFs. However, *TREM2* p.R47H is almost absent in East Asians^8,9^, suggesting that it might also be extremely rare in Native Americans. For the rest of the variants, we performed a search in ExaC database (exac.broadinstitute.org), and found that *PLCG2* p.P522R, and *TREM2* p.R62H were not detected in approximately 4300 East Asian individuals. Unfortunately, results for *ABI3* p.S209F are not reliable due to low quality. Notwithstanding, these observations, together with our results showing that the chromosomes containing these rare variants have mainly Caucasian ancestry in the identified carriers, strongly suggest a European origin for these variants. However, the possibility still remains open that the loci containing the studied rare variants might have an ancestry other than Caucasian. To answer this question, additional studies comparing SNPs among ancestral populations are needed to identify AIMs that better explain the ancestry of these loci.

In conclusion, we report the first genetic data from Argentinian population, which support the contribution of rare coding variants to AD susceptibility. Although this population size is not enough to reach statistical significance for the rare variants studied here, it is a relevant opportunity to start filling the gap on AD genetic architecture in Latin American admixed populations. Our analysis fosters further analysis of these rare variants in other Latin populations to confirm our initial observation. Importantly, expanding research to admixed populations, like this one, will help to identify potential population-specific effects on the genetic structure of AD, in addition to better define conserved relevant pathways involved in the disease.

## Supplementary information

Supplemetal Figures

## References

[1] Gatz M (2006). Role of genes and environments for explaining Alzheimer Disease. Arch. Gen. Psychiatry.

[2] Naj AC, Schellenberg GD (2017). Genomic variants, genes, and pathways of Alzheimer’s disease: an overview. Am. J. Med Genet. B Neuropsychiatr. Genet..

[3] Del-Aguila JL (2015). Alzheimer’s disease: rare variants with large effect sizes. Curr. Opin. Genet. Dev..

[4] Sims R (2017). Rare coding variants in PLCG2, ABI3, and TREM2 implicate microglial-mediated innate immunity in Alzheimer’s disease. Nat. Genet..

[5] Benitez Ba (2013). TREM2 is associated with the risk of Alzheimer’s disease in Spanish population. Neurobiol. Aging.

[6] Finelli D (2015). TREM2 analysis and increased risk of Alzheimer’s disease. Neurobiol. Aging.

[7] Jin SC (2015). TREM2 is associated with increased risk for Alzheimer’s disease in African Americans. Mol. Neurodegener..

[8] Miyashita A (2014). Lack of genetic association between TREM2 and late-onset Alzheimer’s disease in a Japanese population. J. Alzheimers Dis..

[9] Huang M (2015). Lack of genetic association between TREM2 and Alzheimer’s disease in East Asian population. Am. J. Alzheimer’s Dis. Other Dement..

[10] McKhann GM (2011). The diagnosis of dementia due to Alzheimer’s disease: recommendations from the National Institute on Aging-Alzheimer’s Association workgroups on diagnostic guidelines for Alzheimer’s disease. Alzheimer’s Dement..

[11] Viechtbauer W (2010). Conducting meta-analyses in R with the metafor package. J. Stat. Softw..

[12] Anderson CA (2010). Data quality control in genetic case-control association studies. Nat. Protoc..

[13] Chang CC (2015). Second-generation PLINK: rising to the challenge of larger and richer datasets. Gigascience.

[14] R Core Team. *R: A Language and Environment for Statistical Computing* (R Foundation for Statistical Computing, Vienna, 2018).

[15] Galanter JM (2012). Development of a panel of genome-wide ancestry informative markers to study admixture throughout the americas. PLoS Genet..

[16] Alexander DH, Novembre J, Lange K (2009). Fast model-based estimation of ancestry in unrelated individuals. Genome Res..

[17] Morelli L, Leoni J, Castano EM, Mangone CA, Lambierto A (1996). Apolipoprotein E polymorphism and late onset Alzheimer’s disease in Argentina. J. Neurol. Neurosurg. Psychiatry.

[18] Avena S (2012). Heterogeneity in genetic admixture across different regions of argentina. PLoS One.

[19] Bodner M (2012). Rapid coastal spread of First Americans: novel insights from South America’s Southern Cone mitochondrial genomes. Genome Res..

[20] Devoto FJ (1989). Argentine migration policy and movements of the European population (1876-1925). Estud. Migr. Latinoam..

[21] Jacquier M (2001). APOE e4 and Alzheimer’s disease: positive association in a Colombian clinical series and review of the Latin-American studies. Arq. Neuropsiquiatr..

[22] Ward A (2012). Prevalence of Apolipoprotein E4 genotype and homozygotes (APOE e4/4) among patients diagnosed with Alzheimer’s disease: a systematic review and meta-analysis. Neuroepidemiology.

[23] Henderson JN (2002). Apolipoprotein E4 and tau allele frequencies among Choctaw Indians. Neurosci. Lett..

