# Supplementary material for: Transethnic meta-analysis of rare coding variants in PLCG2, ABI3, and TREM2 supports their general contribution to Alzheimer’s disease
Source: Transl Psychiatry. 2019 Jan 31;9:55. doi: 10.1038/s41398-019-0394-9 (PMC6355764; doi:10.1038/s41398-019-0394-9)
Supplement: Supplementary file 1 — Supplemetal Figures [file 41398_2019_394_MOESM1_ESM.pdf]

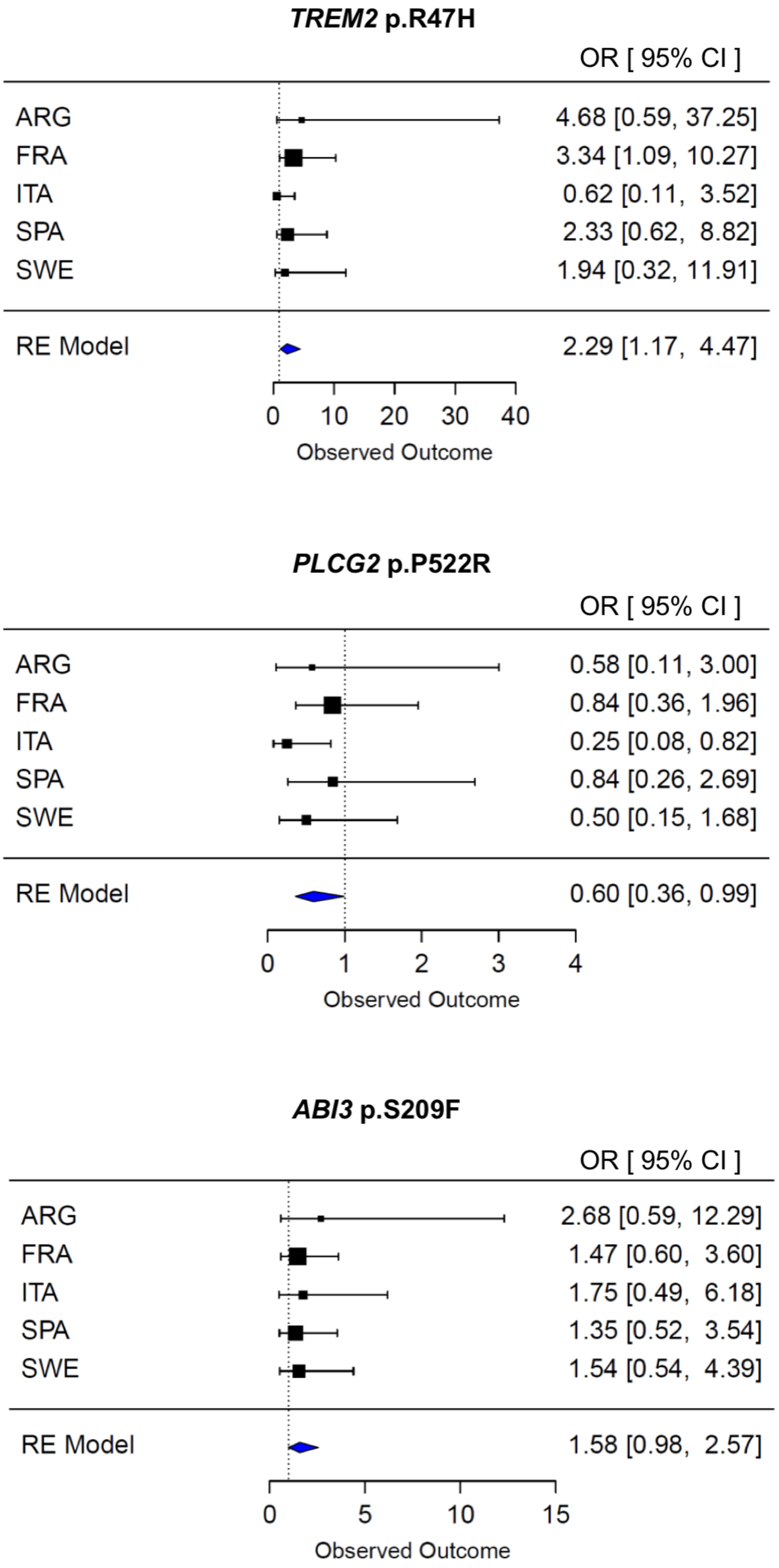

Figure S1. Meta-analysis of ARG and EADI samples. A random-effects (RE) model was used, which assumes that observed effect can vary across studies. ARG, Argentina; FRA, France; ITA, Italy; SPA, Spain; SWE, Sweden. Squared size represents population sample size.

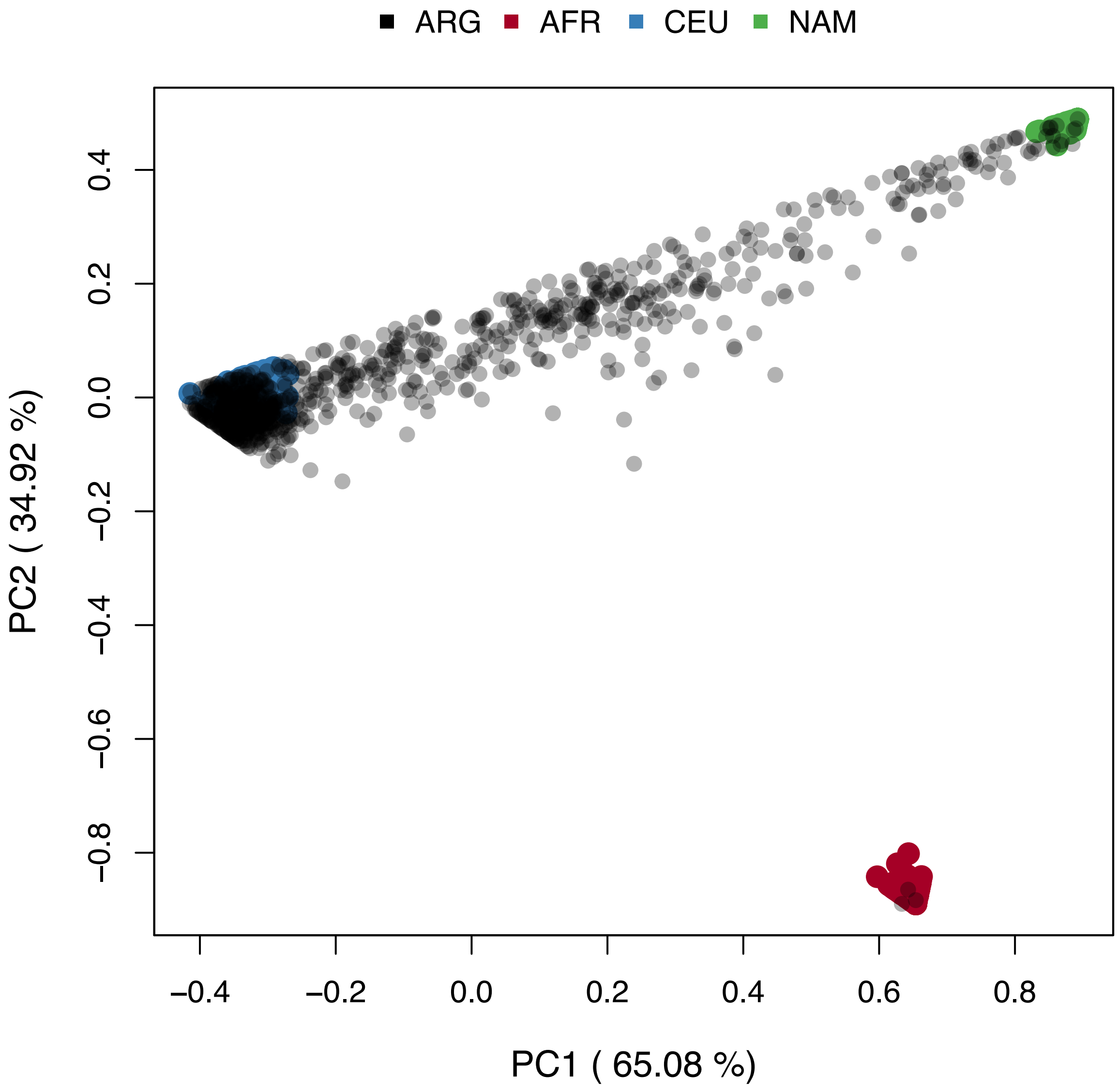

Figure S2. Ancestry of population. Principal component analysis of ancestry results. Black circles, Argentinian individuals (ARG); green circles, Native Americans (NAM); red circles, Africans (AFR); blue circles, Europeans (CEU). Percent of distribution explained by each principal component (PC) it is showed between parenthesis.

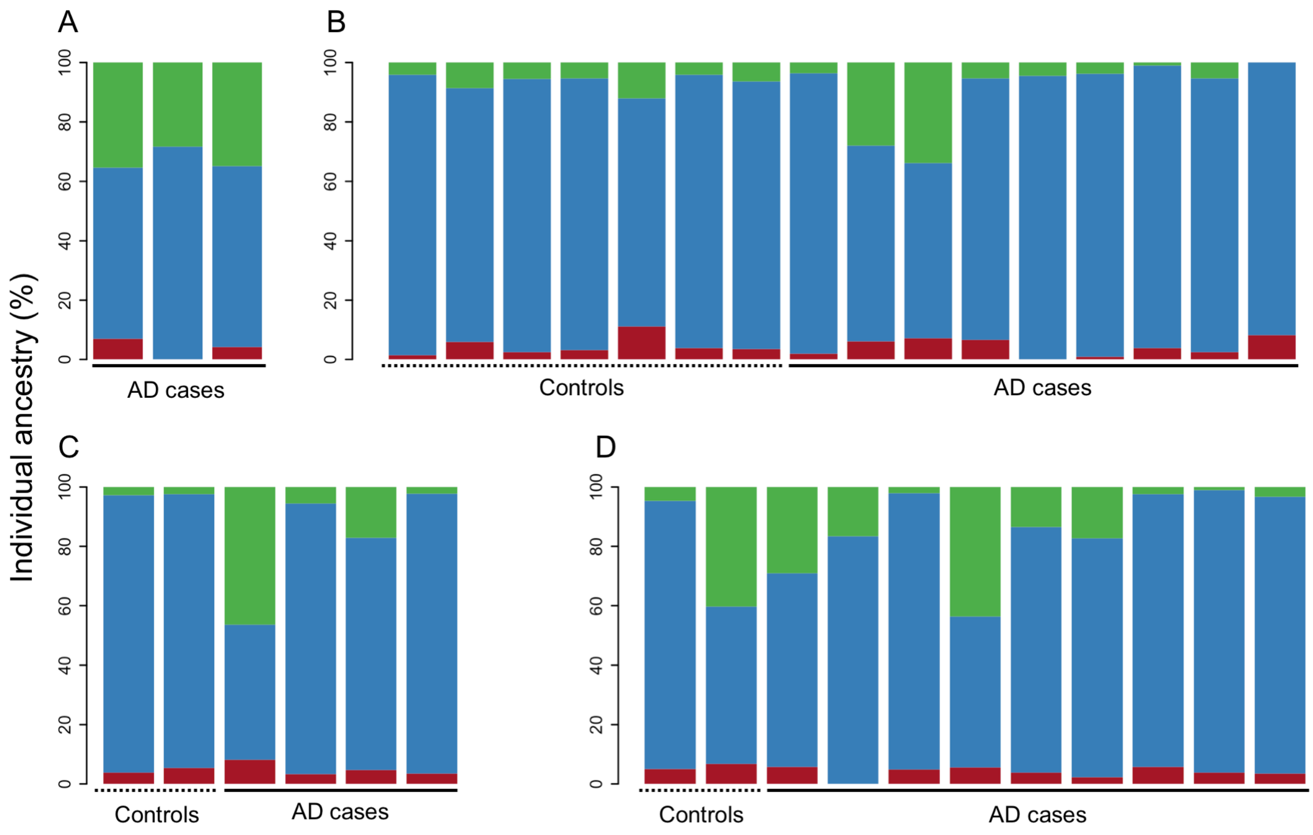

Figure S3. Individual ancestry proportion of people carrying a rare variant. Bar-plots representing each individual on the x-axis, and his percent of European (blue), African (red), and Native American (green) ancestry on the y-axis. **A.** *TREM2* p.R47H carriers. **B.** *TREM2* p.R62H carriers. **C.** *PLCG2* p.P522R carriers. **D.** *ABI3* p.S209F carriers.
